# Cross-sectional and prospective study on anti-Müllerian hormone changes in a cohort of pre-menopausal women with a history of differentiated thyroid cancer

**DOI:** 10.1186/s13044-020-0075-z

**Published:** 2020-01-10

**Authors:** Miranda Mittica, Andrea Dotto, Martina Comina, Marsida Teliti, Eleonora Monti, Massimo Giusti

**Affiliations:** 1 Endocrine Unit, Policlinico San Martino Hospital, Genoa, Italy; 2 Dipartimento di Medicina Interna e Specialità Mediche, University of Genova, Viale Benedetto XV, n° 6, I-16100 Genoa, Italy

**Keywords:** AMH, Radioiodine, Thyroid cancer, Pre-menopausal women

## Abstract

**Background:**

Anti-Müllerian hormone (AMH) decreases acutely after post-surgical radioactive iodine (RAI) ablation in females with differentiated thyroid cancer (DTC).

**Aim:**

We performed a cross-sectional and prospective study on AMH levels in pre-menopausal females with a history of DTC.

**Methods:**

Fifty-nine females after surgery and RAI (group 1) and 30 females after surgery alone (group 2) were studied. The control group consisted of 141 healthy women (group 3). The prospective study was performed in 43 and 14 females from groups 1 and 2, respectively.

**Results:**

On first evaluation, AMH levels were similar in groups 1 and 2, but lower than in group 3. In all groups, AMH was negatively related with chronological age and FSH levels. When subjects were stratified according to age, AMH levels were not different between groups. When AMH was evaluated up to 2 years after the baseline evaluation, no changes emerged in either group of women with DTC. In the prospective study, the incidence of abnormal menstrual cycles and the onset of menopause were observed in similar percentages of women with a history of RAI-treated DTC and of those treated with surgery alone.

**Conclusions:**

AMH can be considered a reliable index of ovarian reserve in women with DTC. Chronological age is the main factor influencing AMH levels in both DTC patients and controls. After age-related stratification, AMH levels are similar in women with DTC treated with RAI and those treated with surgery alone.

## Introduction

Thyroidectomy is the gold standard for the primary treatment of differentiated thyroid cancer (DTC). Subsequent radioactive iodine (RAI) ablation of the thyroid remnant is currently recommended only in selected cases: patients with tumors < 2 cm and distant metastases, and in those with tumors > 2 cm and one of the following risk factors: gross extra-thyroidal extension, age > 45 years, lymph node and distant metastases [1, 2]. Moreover, the literature data indicate an overuse of RAI in low-risk DTC patients [3]. When RAI is administered in young pre-menopausal females, they are advised not to conceive for 6–12 months after treatment; ovarian exposure is estimated to be 140 mGy for an administered activity of 100 mCi [4]. RAI is recognized as being a common cause of transient menstrual dysfunction, accompanied by an increased serum gonadotropin concentration in up to 27% of women [5]. In addition, in RAI-treated women, the literature data report an increased number of miscarriages [6] and a higher incidence of early menopause [5, 7–9].

The measurement of anti-Müllerian hormone (AMH), which is produced and secreted by ovarian granulosa cells, reflects the pool of small growing follicles and is an index of the “ovarian reserve” [10, 11]. AMH shows a longitudinal decline over time, after peaking in the mid-twenties, and reflects the age-related decline of the ovarian follicle pool better than other ovarian reserve markers [12, 13]. AMH predicts the ovarian response during medically assisted pregnancy, the timing of menopause, and iatrogenic damage to the ovarian follicle reserve [11]. To our knowledge, only 4 papers on AMH in DTC females have so far been published. First, in 45 women aged 36 years on average who had been exposed to RAI, Acibucu et al. [14] reported lower AMH levels than in 40 healthy body mass index (BMI)- and age-matched females. We [15] measured AMH levels in 34 pre-menopausal women treated with RAI for DTC and compared these with those of 23 age-matched women with DTC who had not received RAI therapy. AMH levels were only slightly, and not significantly, lower in those patients who had undergone RAI [15]. In both studies, the interval between thyroidectomy and RAI administration and AMH sample collection was of several months: more than 36 months in the Acibucu et al. study [14] and 5 years on average in our study [15]. Recently, Yaish et al. [16] and Evranos et al. [17] designed similar studies in which AMH was evaluated before and 3–12 months after RAI administration in DTC females. These studies showed a significant decrease in AMH levels, with lower levels 3 months after RAI, and an increasing trend in the following months, though pre-RAI levels were not reached by the 12-month evaluation. On the other hand, in a very small number of females who underwent low RAI activity for Graves’ disease, no changes in AMH levels were detected [16].

Moreover, at present, while the acute effects of RAI on AMH levels in DTC females can be considered well documented [16, 17], the long-term effects are not well defined [14, 15]. Small numbers of patients and controls, differences in age between study groups, differences in RAI activities administered to DTC patients, inadequate follow-up, and differences in the sensitivity of AMH assays suggest that further studies are needed in this field.

The aim of the present study was to increase the number of AMH evaluations in pre-menopausal females with a history of DTC, treated or not with RAI, and to start a prospective AMH evaluation by collecting additional samples from the same patients when no further RAI treatment was given. Since a limitation of our previous study [15] was the lack of a control group of normal pre-menopausal women with normal thyroid function and the lack of in-house age-related AMH reference values, in the present study a large group of normal pre-menopausal females was recruited in order to ascertain whether AMH changes in DTC were RAI- and thyroid pathology-related or age-related. Chronological age, BMI, therapies, menstrual cycle characteristics, pregnancies, pituitary-gonadal hormones, TSH and f-T4 levels were considered. The primary objective was to evaluate long-term effects of RAI exposure on AMH and ovarian function in pre-menopausal DTC females; the secondary objective was to obtain normative AMH values in DTC females, as there are already available in normal control women stratified for age.

In an era in which all guidelines discourage the use of RAI in very low- and low-risk thyroid cancer [18], a long-term evaluation of damage to the ovarian reserve, as measured through AMH levels, could further suggest caution in the use of RAI in women of reproductive age.

## Materials and methods

### Subjects

Eighty-nine pre-menopausal women aged over 18 years who were not on hormonal contraception and who had a history of DTC were recruited in 2017–19 during their annual follow-up examinations at our out-patient Thyroid Cancer Unit. The majority of thyroid cancers were papillary thyroid cancer (PTC, 86% of total), while in the remaining cases histology revealed a follicular variant of PTC (7%) or follicular thyroid carcinoma (7%). All cancers were low-risk thyroid cancer on diagnosis, according to the 8th AJCC TNM staging system. Women treated with chemotherapy or external beam radiation for a secondary tumor were excluded. All subjects were on long-term L-T4 treatment to achieve age- and risk-adjusted TSH levels [18]. In all patients, lobectomy or total thyroidectomy had been performed as the primary treatment 6.7 years (median 5 years; range: 1–32 years) before the present study. Biochemical disease, without evidence of structural disease, was observed in two women. Patients were subdivided according to whether they had previously undergone RAI ablative treatment (group 1; *n* = 59) or not (group 2; *n* = 30). Pre-menopausal women with normal menstrual cycles and without a history of neck surgery or radiation (*n* = 141) served as a control group (group 3). DTC females and controls were arbitrarily divided into subgroups according to age (18–25, 26–30, 31–35, 36–40, 41–45, > 46 years). Some clinical data are reported in Table 1.

**Table 1:** Some clinical data on the groups of subjects studied. Median and 25th and 75th percentile values are reported in brackets

	Group 1 *n* = 59	Group 2 *n* = 30	Group 3 *n* = 141	Significance *P*
Age (years)	41.2 ± 7.5 (43; 37–47)	42.4 ± 9.2 (45; 40–49)	33.1 ± 10.1 (33; 24–42)	< 0.0001; a
Mother’s age on menopause (years)	51.0 ± 4.2 (51; 50–54)	51.1 ± 4.2 (51; 49–55)	50.1 ± 4.2 (51; 48–52)	0.57
Time since primary treatments (years)	7.2 ± 6.8 (5: 2–9)	4.6 ± 4.1 (3; 1–7)	n.a.	0.07
BMI (kg/m2)	25.3 ± 6.0 (23.5; 21.6–27.0)	24.2 ± 6.0 (23.5; 21.6–27.0)	24.5 ± 6.5 (23.8; 21.0–25.3)	0.45
Cumulative RAI activity (mCi)	100.1 ± 117.4 (80; 60–100)	n.a.	n.a.	–
Cured (%) / Biochemical disease (%)	97/3	100/0	n.a.	0.55
L-T4 dosage (μg/week)	859.1 ± 157.3 (825; 775–925)	741.3 ± 148.4 (725; 644–875)	n.a.	0.001
TSH (mIU/l)	0.46 ± 0.83 (0.17; 0.04–0.42)	1.69 ± 1.78 (1.37; 0.25–2.31)	2.35 ± 1.79 (2.11; 1.23–2.81)	< 0.0001; b, c
f-T4 (pmol/l)	20.5 ± 4.9 (20.7; 18.1–23.2)	16.5 ± 3.9 (16.4; 15.2–19.0)	13.8 ± 3.3 (13.4; 11.4–16.7)	< 0.0001; d, e, f
Tg (μg/l)	0.24 ± 0.47 (0.20; 0.04–0.20)	1.32 ± 3.20 (0.20; 0.09–0.67)	n.d.	0.01
TgAb positive (%)	2	4	n.d.	0.17

(When ANOVA was significant, differences among groups were evaluated by means of Dunn’s test. Age: (a) group 3 vs group 1 and group 2, both *P* < 0.0001. TSH: (b) group 1 vs group 2, *P* = 0.03; (c) group 1 vs group 3, *P* < 0.0001. Free-T4: (d) group 1 vs group 2, *P* = 0.003; (e) group 1 vs group 3, *P* < 0.0001; (f) group 2 vs group 3, *P* = 0.003. Evaluation not applicable: *n.a.*; evaluation not done: *n.d.*)

Forty-three DTC females from group 1 and 14 from group 2 were evaluated prospectively after the first examination. Written informed consent to participate in the study was obtained from all patients.

### Protocol

The inclusion criterion was the presence of spontaneous menses at the time of the first examination. Clinical examinations included neck ultrasonography, body weight evaluation and pharmacological anamnesis. The mother’s age on spontaneous menopause, characteristics of the menstrual cycle, details regarding previous pregnancies, miscarriages and parity were recorded. In addition to the tests for monitoring patients on L-T4 therapy, and for monitoring the disease, two additional blood samples were requested: one in the early follicular phase (day 2–3 of the menstrual cycle) for AMH, estradiol and FSH evaluation, and one in the luteal phase (day 21–24 of the menstrual cycle) for prolactin (PRL) and progesterone evaluation. With the exclusion of thyroglobulin (Tg) and Tg-antibodies (TgAb), the same evaluations were performed in group 3 women. All samples were drawn in the morning in the fasting condition.

### Assays

Serum AMH was measured by means of a fully-automated two-site immunoassay, as previously reported [15]. The functional sensitivity is 0.21 pmol/l. The expected values in pre-menopausal women on day 2–3 of the menstrual cycle range from 9.3 to 104.3 pmol/l. FSH, estradiol, PRL and progesterone were measured by means of enzyme-enhanced chemiluminescent immunoassays. The expected values of FSH and estradiol in the early follicular phase are less than 14.4 mIU/l and 308.7 pmol/l., respectively. The expected values of PRL and progesterone in the mid-luteal phase are less than 25 μg/l and more than 17 nmol/l, respectively. Free-T4 (f-T4; reference range 12.0–22.0 pmol/l), TSH (reference range 12.0–22.0 pmol/l; functional sensitivity 0.01 mIU/l), Tg (functional sensitivity 0.1 μg/l), and TgAb were evaluated as previously reported [19].

### Statistical analysis

Menstrual cycles with an interval of 28 ± 2 days and 3–5 days of bleeding were deemed regular; changes in bleeding intervals (oligomenorrhea or polymenorrhea) and flow (hypomenorrhea, hypermenorrhea) were scored according to Read & Carr [20]. On the basis of the functional sensitivity of the Tg assays, group 1 DTC females with undetectable Tg levels, negative TgAb and negative neck sonography were considered disease-free; group 2 DTC females. in whom Tg levels were low but detectable or stable over time and neck US imaging was negative were also considered to be disease-free.

The GraphPad 8.3 software (GraphPad, San Diego, CA, USA) was used for statistical analysis. To compare continuous data, the Kruskal-Wallis analysis of variance (ANOVA), followed by Dunn’s multiple comparisons test, and the Mann-Whitney test and the Wilcoxon test were used. Percentages were compared by means of Fisher’s exact test. Correlations were evaluated by means of the Spearman test. Data are reported as mean ± standard deviation (SD). Significance was set at *P* < 0.05. Data collection and subsequent analysis were performed in compliance with the Helsinki Declaration.

## Results

### Cross-sectional study

Table 1 shows some clinical data of the subjects studied. Chronological age differed among the three groups of women, being higher in those with DTC than in controls. BMI and the mother’s age on the appearance of menarche were similar in the three groups of women. TSH was significantly lower in group 1 than in group 2, while f-T4 was significantly higher. Likewise, levels of TSH were lower in group 1 than in group 3. In group 3, the average value of f-T4 was lower than that observed in women receiving L-T4 therapy for a history of DTC. Tg levels were significantly lower after RAI than after thyroidectomy alone, while the numbers of DTC women cured of thyroid cancer or with TgAb positivity were comparable (Table 1).

Owing to age differences among the groups, the number of women who had had full-term pregnancies differed: group 1 (62%), group 2 (47%) and group 3 (23%) (*P* < 0.0001). Six full-term pregnancies occurred after RAI. The rate of abortions was 14, 13 and 22% in groups 1, 2 and 3, respectively (*P* = 0.28) and was similar in women with DTC who had undergone RAI treatment and in those who had not (*P* = 0.99). In group 1, no miscarriage was reported after RAI.

At a similar time after primary treatments (Table 1), AMH was not significantly different between the two groups of women with a history of DTC (Table 2). Estradiol, progesterone and PRL did not show differences among the three groups of subjects, whereas FSH was significantly (*P* = 0.01) lower in group 3 than in group 1.

**Table 2:** AMH and pituitary-gonadal hormones (median ± SD) observed during the menstrual cycle; see also *protocol* section. Median and 25th and 75th percentile values are reported in brackets

	Group 1 *n* = 59	Group 2 *n* = 30	Group 3 *n* = 141	Significance *P*
AMH (pmol/l)	9.79 ± 11.46 (7.14;1.57–12.29)	8.71 ± 10.54 (3.75;1.48–16.13)	21.28 ± 23.82 (13.50; 5.14–29.32)	< 0.0001; a
FSH (IU/l)	12.6 ± 12.4 (8.6; 6.2–13.8)	15.3 ± 15.4 (11.0; 6.6–17.9)	10.1 ± 12.0 (6.8; 4.9–10.2)	0.005; b
Estradiol (pmol/l)	210.8 ± 87.6 (176.2; 147–247)	162.8 ± 65.9 (168.9; 109–209)	204.1 ± 171.3 (157.9;105–209)	0.10
Progesterone (nmol/l)	22.2 ± 18.1 (22.3; 4.8–33.5)	25.2 ± 16.6 (23.4; 14.9–36.4)	23.0 ± 24.2 (19.5; 3.7–30.2)	0.64
PRL (μg/l)	12.3 ± 8.5 (9.6; 6.3–16.0)	13.8 ± 6.6 (13.7; 7.6–17.7)	14.8 ± 8.4 (13.3; 8.5–18.6)	0.06

(When ANOVA was significant, differences among groups were evaluated by means of Dunn’s test. a) AMH: group 3 vs group 1, *P* = 0.0002 and group 3 vs group 2, *P* = 0.001; b) FSH: group 3 vs group 1, *P* = 0.01; group 3 vs group 2, *P* = 0.06)

**Fig. 1:**
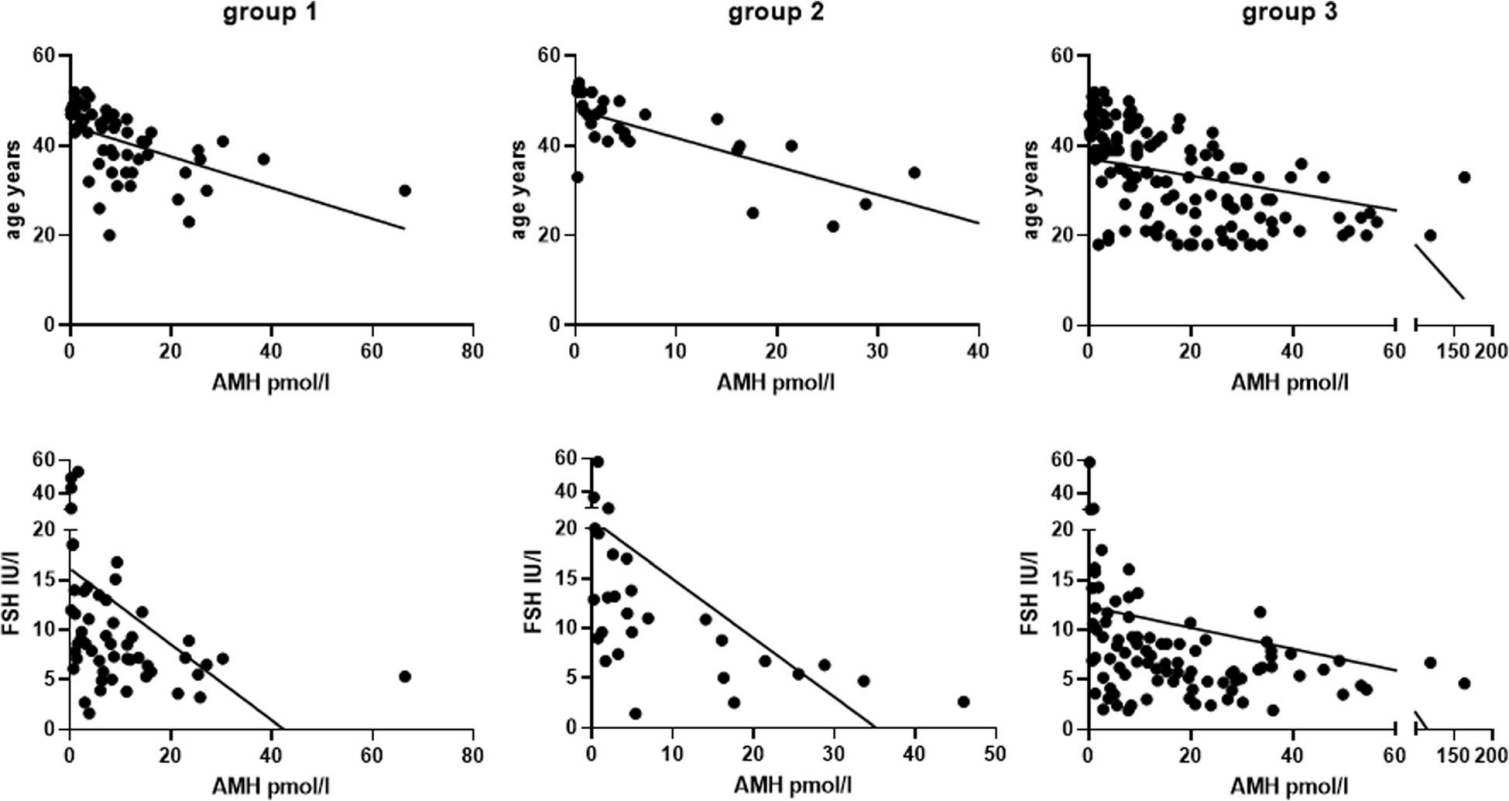
Correlations between AMH and chronological age (top) or FSH levels (bottom) in the three groups of pre-menopausal females studied. For significance, see also Table 3

**Fig. 2:**
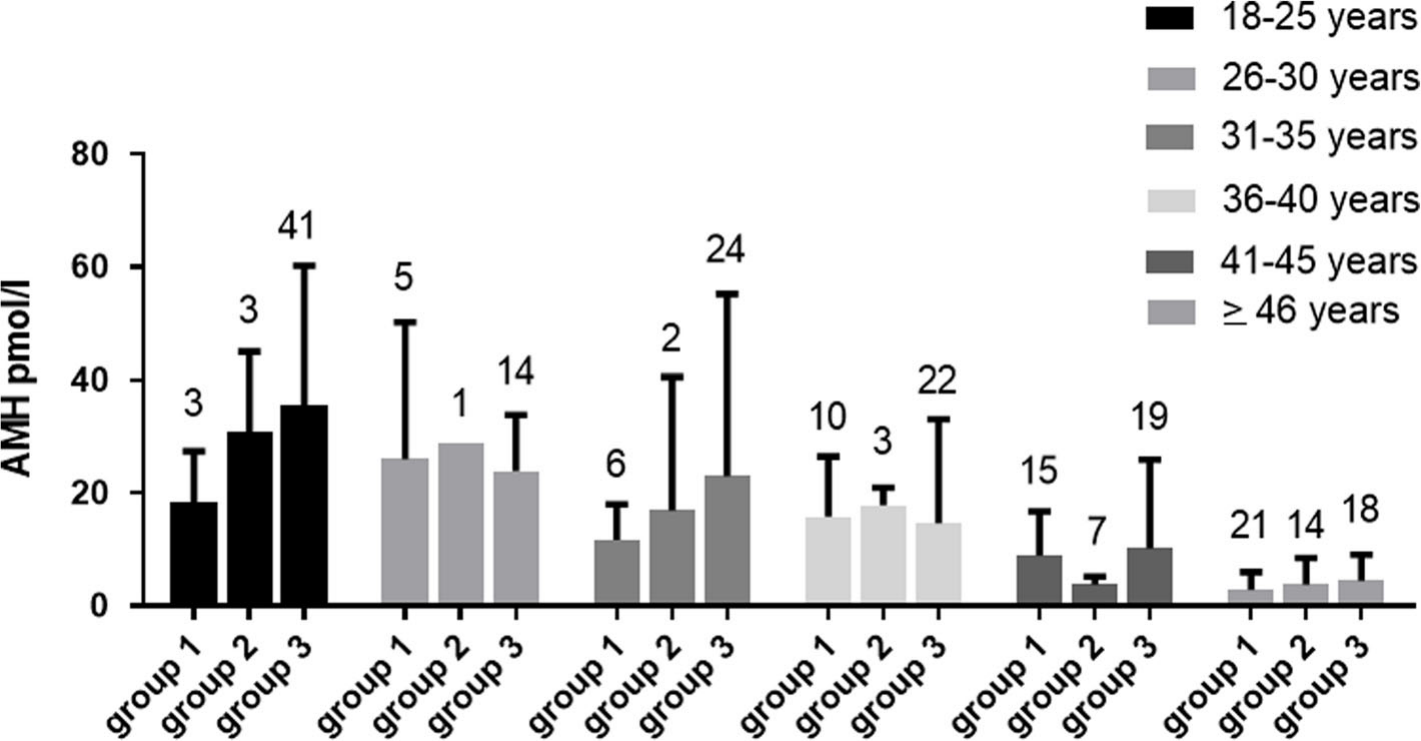
Mean (± SD) AMH levels observed in the three groups of pre-menopausal females stratified in quintiles according to chronological age. The number at the top of each column indicates the number of subjects in each quintile

In the three groups of women, there was a significant (*P* < 0.0001) inverse relationship between AMH and age and between AMH and FSH levels (Fig. 1). Table 3 shows the data regarding the correlations between AMH and the other clinical-laboratory parameters evaluated. In Fig. 2, the average values of AMH are stratified according to age in the three groups of women. On ANOVA, no differences in the mean values of AMH among the three groups of women were seen (Fig. 2).

**Table 3:** Correlation between AMH levels and some clinical and biochemical parameters. The number of pairs evaluated is reported in brackets

	Group 1		Group 2		Group 3	
	Sr	*P*	Sr	*P*	Sr	*P*
Age	−0.71 (59)	< 0.0001	− 0.72 (30)	< 0.0001	− 0.62 (141)	< 0.0001
Years since diagnosis	− 0.20 (59)	0.14	0.03 (30)	0.89	n.a.	n.a.
RAI activity	−0.11 (59)	0.38	n.a.	n.a.	n.a.	n.a
BMI	0.02(59)	0.84	0.05 (30)	0.81	0.01 (132)	0.93
L-T4 dosage/week	0.13 (59)	0.32	−0.11 (30)	0.55	n.a	n.a
TSH	−0.04 (59)	0.78	0.16 (30)	0.40	0.14 (121)	0.12
f-T4	0.06 (59)	0.65	0.32 (30)	0.09	−0.13 (105)	0.19
Thyroglobulin	−0.05 (59)	0.78	0.04 (30)	0.81	n.a	n.a.
FSH	−0.55 (56)	< 0.0001	−0.77 (30)	< 0.0001	− 0.46 (102)	< 0.0001
Estradiol	−0.13 (54)	0.35	0.20 (28)	0.30	−0.16 (98)	0.12
Progesterone	0.28 (52)	0.05	0.28 (26)	0.16	−0.18 (85)	0.09
PRL	0.09 (57)	0.52	0.09 (26)	0.68	−0.11 (97)	0.26

*n.a.* Not applicable

### Prospective evaluation

**Fig. 3:**
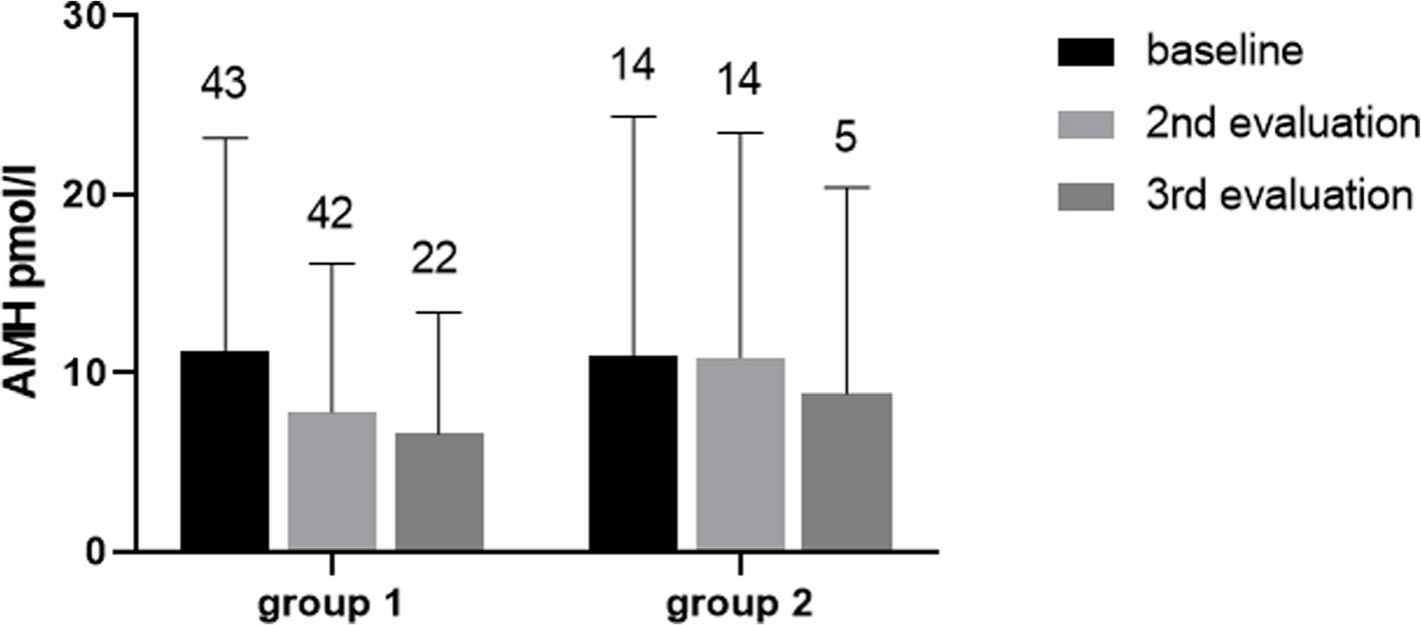
Mean (± SD) AMH levels observed in group 1 and group 2 from baseline to 3rd evaluation. The number at the top of each column indicates the number of subjects at each examination

Data were available in 43 women in group 1 and 14 in group 2. At the baseline examination, age was similar (*P* = 0.35) in group 1 [40.8 ± 6.8 years (median 43.0 years; range 23–52 years)] and group 2 [41.3 ± 10.3 years (median 46.0 years; range 20–54 years)]. Two subsequent AMH evaluations were performed after 15.0 ± 5.6 months and 31.2 ± 10.6 months in group 1 and after 18.6 ± 9.6 months and 27.4 ± 6.3 months in group 2. In group 1, AMH data were not available owing to: follow-up < 12 months in 3 cases, menopause in 5 cases, pregnancy in 1 case, treatment with hormonal contraceptive in 1 case, and non-adherence to the protocol in 6 cases. In group 2, prospective AMH data were not available owing to: follow-up < 12 months in 2 cases, menopause in 4 cases, pregnancy in 1 case, treatment with hormonal contraceptive or long-acting LHRH in 2 cases, initiation of radiometabolic treatment initially not planned in 1 case, and non-adherence to the protocol in 6 cases. During the subsequent observation in group 1, menopause occurred in another 4 women, and pregnancy in 2 women, one of whom miscarried. Dysfunctional cycles were documented in 36% of women with spontaneous cycles. In group 2, menopause occurred in another woman during follow-up. Dysfunctional cycles were documented in 30% of women with spontaneous cycles. Overall, the onset of menopause was observed in similar percentages of women with a history of RAI-treated DTC (21%) and of those untreated with RAI (17%; *P* = 0.76). Figure 3 shows the average trend of AMH in the two groups of subjects. In both groups, a non-significant reduction in AMH was detected on ANOVA (group 1: *P* = 0.14; group 2: *P* = 0.88). However, on applying Wilcoxon’s paired test in order to compare only the data obtained in the first two evaluations, AMH significantly (*P* < 0.001) decreased between the first (*n* = 42; median 8.57 pmol/l; 25th–75th percentile 2.96–14.48 pmol/l) and the second evaluation (5.42 pmol/l; 1.05–36.92 pmol/l) only in group 1. No significant variations were observed between the first and the second determination in group 2 (Fig. 3).

## Discussion

AMH is a marker of gonadal reserve [10, 11]. In pre-menopausal females, the population of ovarian follicles of 5–8 mm diameter produces most of the circulating AMH [10]. Unlike other markers of ovarian reserve, such as FSH and inhibin B, AMH has little intra-cycle and inter-cycle variability, which makes it an easier marker to use in evaluating ovarian reserve [11]. As AMH shows a gradual age-related decline, age-specific ranges are required for its evaluation; after menopause, AMH is undetectable [11–13]. In the present study, we collected samples from a large group of control females without thyroid pathology, in order to obtain age-specific AMH ranges. When AMH was stratified in quintiles according to chronological age, we confirmed that AMH declined after 30 years of age, sometimes reaching undetectable levels after 45 year of age.

At least a third of women who receive a diagnosis of DTC are less than 45 years old and want to know whether their cancer treatment will contribute to the natural decline of their ovarian reserve. Roberts et al. [21] studied AMH in young females who were survivors from various cancers, including a large cohort of subjects with a history of thyroid/skin cancer. They documented a wide range of AMH levels, from 3.5 pmol/l to 29.8 pmol/l, which was regarded as a reference range because, in these cancers, therapies were not thought to be toxic to ovaries [21]. In our previous paper [15], pre-menopausal females with a history of low-risk DTC who underwent RAI treatment had mean AMH levels of 10.7 pmol/l, which were only slightly, and not significantly, lower than those (17.5 pmol/l on average) observed in age-matched DTC females who had not undergone ablative RAI. Limitations of that study were the small size of the sample population, the absence of AMH date near to RAI treatment, and the difference in the time interval from the DTC diagnosis to AMH evaluation [15]. These difficulties remain in the present paper and reflect a less generalized use of RAI in low-grade DTC [18]. In addition, risk-related TSH suppression is now suggested in DTC patients who do not need RAI ablation [18]. These current strategies for the management of DTC patients can explain the differences observed in L-T4 dosages and levels of TSH, Tg and f-T4 among the groups of DTC females studied.

AMH shows an age-related decline in DTC women ([15], present study) as in normal women ([10, 11], present study). On stratifying women with DTC into two groups, Evranos et al. [16] observed that AMH values were significantly lower in women > 30 years than in those aged < 30 years. Acibucu et al. [14] found that AMH levels in a group of 45 women with DTC aged 35 years on average who had undergone RAI treatment were lower than in healthy control females matched for age and BMI. The authors considered this difference to be a consequence of the increase in hormone clearance due to exogenous hyperthyroxinemia [14]. More recently, two groups of researchers evaluated AMH before and after a single RAI treatment [16, 17]. AMH declined after RAI, reaching a nadir 3–6 months later and then subsequently increasing, though without reaching pre-treatment levels 12 months later [16, 17]. These two studies, however, were limited by the low number of patients involved, inter-patient differences in the RAI activity administered and the unclear modality of preparation for ablative treatment [16, 17]. Moreover, in the study by Yaish et al. [16], the control group consisted of a very small group of subjects treated with < 30 mCi of RAI for hyperthyroidism, in whom the time-course of AMH did not show changes [16]. However, none of the available studies found a correlation between the RAI activity administered and AMH levels/decrease [15–17]; this might be due to individual sensitivity to RAI, though a much larger number of women would need to be studied in order to obtain conclusive data in this field.

Previously, we observed a high degree of menstrual abnormalities in RAI-treated women [15]. In the present study, during longitudinal evaluation of AMH levels, we recorded a dysfunctional menstrual cycle in some women with DTC without contraceptive treatment, whether or not they had undergone RAI treatment. This finding, however, could be due more to the progression of chronological age than to DTC and its treatment. This observation of ours seems to be in contrast with Acibucu et al. [14], who reported, as an acute effect of RAI, oligo/amenorrhea in 16% of patients at least 3–5 months after treatment. Evranos et al. [17] did not find changes in LH, FSH and estradiol levels after RAI. Although we previously reported a positive correlation between AMH and progesterone [15], in the present study we did not find differences in estradiol and progesterone levels in our three groups of women evaluated. Data are available on the relationship between AMH and spontaneous pregnancy. In young women with low AMH, fertility is not compromised [22] and in females aged 30–44 years no relationship between low AMH and pregnancy rates has been reported [23]. However, AMH is used to predict the rate of success in controlled ovarian stimulation, and a cut-off AMH level of 7.71 pmol/l, especially to predict poor responders, has been reported [24]. Acibucu et al. [14] reported that 18% of women became pregnant after RAI treatment, and that no patient who wanted to become pregnant failed to do so. Our previous study documented a similar pregnancy or miscarriage rate in women with DTC, whether treated with RAI or not [15]. The present data confirmed this observation, and the cumulative beginning of menopause was similar in both groups (group 1: 21%; group 2: 17%). Overall, we think that RAI does not influence menstrual cycle characteristics and reproduction, which are phenomena that are more age-related. The finding of a two-figure rate of abortions in our control group (22%) does not surprise us, as it was due to voluntarily terminated pregnancies in females of young age. FSH and estradiol reflect ovarian activity in the early follicular phase. Anderson et al. [25] found a negative correlation of AMH with FSH, but not with estradiol levels. Our data confirm an inverse relationship between FSH and AMH in both in controls and in all groups of women with DTC. Like AMH, FSH reflects ovarian activity in pre-menopausal DTC patients and in controls.

Not many studies on AMH and thyroid function are available. After adjustment for thyroid autoimmunity and age, TSH < 3.0 mIU/l in infertile euthyroid patients has been associated with significantly higher AMH than in patients with TSH ≥3.0 mIU/l [26]. Moreover, higher levels of thyroperoxidase antibodies (TPOAb) have been observed in women with a lower ovarian reserve, with a positive trend of these antibodies over time, indicating that this group may be at higher risk of hypothyroidism over time [27]. Kuroda et al. [28] also reported a clear association between elevated TSH and reduced AMH levels. Moreover, L-T4 supplementation in TPOAb-positive patients improves follicular support [29] and prevents miscarriage [30] but does not change average AMH levels [30]. In DTC patients, thyroid autoimmunity, and TgAb in particular, are always evaluated in order to fully judge Tg levels as a marker of cure [18]. In our present study, thyroid autoimmunity was not systematically assessed in control females, while only in a few RAI-untreated DTC females were TgAb positive (4%). In our women with DTC and healthy females, a correlation was not found between TSH and AMH, as previously reported by other researchers [28], and our previous observation [15] of an inverse correlation between L-T4 and AMH was confirmed in RAI-treated DTC women. Overall, these observations suggest that more data on healthy and DTC women are needed in order to define the relationship between thyroid function and AMH levels.

The main limitations of our study, as in the previous one [15], remain the different interval between primary treatments and the first evaluation of AMH, the difficulty of enrolling substantial numbers of women with DTC, whether exposed to RAI or not, in each age-group, and the preliminary significance of the prospective study.

## Conclusions

AMH was confirmed to be a sensitive marker of gonadal function in women with DTC, as in healthy women. The evaluation of AMH can replace the use of FSH in the evaluation of the gonadal reserve in DTC women of fertile age. In both women with DTC and healthy women, age is the factor that most affects the level of AMH. In our limited group of women evaluated at a distance from primary treatments, AMH did not appear to be significantly affected by thyroidectomy and RAI exposure. This finding does not exclude the possibility that, in close proximity to RAI therapy, there is a negative effect on the ovary, as documented in the most recent studies [16, 17]. Therefore, potential gonadal damage must be taken into consideration in therapeutic choices in young women. Currently, in women with DTC, there is a lack pf prospective data in the following areas: AMH values a long time after thyroidectomy; the trend in AMH after the beginning of L-T4 treatment (the dosage of which is often supra-physiologic); and AMH values after ablative/therapeutic use of RAI, when necessary. A more prolonged prospective evaluation conducted on a larger population of DTC women therefore appears indispensable. This need is acutely felt in the light of our current observations of a lower AMH median and a higher percentage of menstrual irregularities in the RAI group. Our prospective study also seems to indicate that, approximately 1 year after the first evaluation, AMH appears to be reduced to a greater degree than would be supposed on the basis of the increase in age. The ultimate aim of assessing ovarian function more accurately through the evaluation of AMH will be to understand whether further caution must be exercised in order to reduce the use of RAI in young women whose desire to procreate has not yet been expressed.

## Data Availability

Data availability from the authors on request.

## Abbreviations

AMH: Anti-Mullerian hormone
DTC: Differentiated thyroid cancer
FSH: Follicle stimulating hormone
F-T4: Free-thyroxine
L-T4: Levo-thyroxine
PRL: Prolactin
RAI: Radiactive iodine
Tg: Thyroglobulin
Tg-Ab: Tg-antibodies
TSH: Thyroid stimulating hormone
